# Effects of Substitution Ratios of Zinc-Substituted Hydroxyapatite on Adsorption and Desorption Behaviors of Bone Morphogenetic Protein-2

**DOI:** 10.3390/ijms231710144

**Published:** 2022-09-04

**Authors:** Baolin Huang, Manchun Li, Hailing Mo, Chuang Chen, Kun Chen

**Affiliations:** The Joint Research Center of Guangzhou University and Keele University for Gene Interference and Application, School of Life Sciences, Guangzhou University, Guangzhou 510006, China

**Keywords:** BMP-2, Zn-HAP, bioactivity, interaction, MD simulation

## Abstract

Understanding interactions between bone morphogenetic proteins (BMPs) and biomaterials is of great significance in preserving the structure and bioactivity of BMPs when utilized in clinical applications. Currently, bone morphogenetic protein-2 (BMP-2) is one of the most important growth factors in bone tissue engineering; however, atomistic interactions between BMP-2 and zinc-substituted hydroxyapatite (Zn-HAP, commonly used in artificial bone implants) have not been well clarified until now. Thus, in this work, the interaction energies, binding/debinding states, and molecular structures of BMP-2 upon a series of Zn-HAP surfaces (Zn-HAPs, 1 at%, 2.5 at%, 5 at%, and 10 at% substitution) were investigated by hybrid molecular dynamics (MD) and steered molecular dynamics (SMD) simulations. Meanwhile, cellular studies including alkaline phosphatase (ALP) activity and reverse transcription-polymerase chain reaction (RT-PCR) assay were performed to verify the theoretical modeling findings. It was found that, compared to pure HAP, Zn-HAPs exhibited a higher binding affinity of BMP-2 at the adsorption process; meanwhile, the detachment of BMP-2 upon Zn-HAPs was more difficult at the desorption process. In addition, molecular structures of BMP-2 could be well stabilized upon Zn-HAPs, especially for Zn10-HAP (with a 10 at% substitution), which showed both the higher stability of cystine-knots and less change in the secondary structures of BMP-2 than those upon HAP. Cellular studies confirmed that higher ALP activity and osteogenic marker gene expression were achieved upon BMP-2/Zn-HAPs than those upon BMP-2/HAP. These findings verified that Zn-HAPs favor the adsorption of BMP-2 and leverage the bioactivity of BMP-2. Together, this work clarified the interaction mechanisms between BMP-2 and Zn-HAPs at the atom level, which could provide new molecular-level insights into the design of BMP-2-loaded biomaterials for bone tissue engineering.

## 1. Introduction

Interactions between proteins and biomaterials have gained increasing attention owing to their widespread occurrence in nature and their broad range of applications in tissue engineering and regenerative medicine [1]. Protein–substrate interactions are vital in preserving the structure and bioactivity of proteins delivered by biomaterials. The characteristics of substrate surfaces, such as topology, wettability, chemical composition, and surface charge, could regulate the final conformation and biological function of the adsorbed proteins [2,3,4]. Protein structures can change from their native state to an unfolded configuration depending on the surface property-guided interactions, which may lead to partial denaturation of the protein [5]. Therefore, deeply understanding the interactions between proteins and biomaterial surfaces is of critical importance to the rational design of new tools in biomaterial sciences.

In bone tissue engineering, bone morphogenetic protein-2 (BMP-2) is an essential protein for new bone formation and restoration because of its multiple potentials including the inducement of bone mesenchymal stem cells into osteoblasts [6,7,8]. In clinic, more and more researchers are utilizing BMP-2-loaded scaffolds in bone tissue engineering, since this strategy can reduce healing time and save medical costs. However, considerable studies have revealed that improper immobilization of BMP-2 could cause changes in molecular conformations and secondary structures and even denaturation of the protein, which affects the biological activity and clinical therapeutic effects of BMP-2 [9,10]. For effective bone regeneration, BMP-2 adsorption upon the scaffold surface must be stable without serious configuration changes and retained exclusively around the bone defect sites. More importantly, the delivered BMP-2 is able to interact with its cellular receptors and to initiate cell signaling cascades [11,12,13]. If the interactions between BMP-2 and substrates are too strong, the BMP-2 molecules may not be able to activate their cellular receptors, which directly affects the bioactivity. Therefore, a balance needs to be achieved between the stable adsorption of protein and the maintenance of protein activity in the functionalization of biomaterial surfaces with BMP-2.

Detailed knowledge of the proteins involved in the adsorption and desorption upon biomaterials, as well as an understanding of the interactions between proteins and substrates, is pivotal for the development of BMP-2-immobilized scaffolds with high efficiency [8,14]. Protein adsorption upon biomaterials at the atom level can be explored by using molecular dynamics (MD) simulations [15,16]. In recent years, the initial adsorption and desorption processes of BMP-2 upon several inorganic biomaterials were investigated by MD simulations. For example, Dong et al. [17,18] demonstrated that the water-bridged H-bond played a significant role in BMP-2–substrate interactions. Similarly, Marquetti et al. [19] reported that the wetting behavior of the dissolution media directly guided the BMP-2–substrate interactions, and thus impacted the secondary structures of BMP-2. It should be noted that the BMP-2 molecule applied in these studies was a monomer, which is not stable in biological form. Utesch et al. [20] utilized dimer proteins of BMP-2 in their MD simulations and observed a weak but stable adsorption of BMP-2 on a hydrophobic graphite surface. In contrast, a loose adsorption of BMP-2 was found on a hydrophilic titanium dioxide surface. In addition, it is known that pure MD simulations need too long a time to investigate the adsorption and desorption processes of large biomolecular systems. To relieve this issue, steered molecular dynamics (SMD) simulations have been developed, which can accelerate the adsorption and desorption processes by applying artificial forces to pull biomolecules toward material surfaces [21,22]. With combined MD and SMD simulations, theoretical modeling of complex processes (such as the unfolding of proteins and structural rearrangement of proteins) became possible on computationally accessible time scales.

As major inorganic components of human bone, hydroxyapatite (HAP) nanocrystals are widely applied in bone tissue engineering. The interactions between BMP-2 and HAP play crucial roles in the bone regeneration process. Recently, several theoretical studies revealed that cation ion (e.g., Mg^2+^, Sr^2+^)-substituted HAP exhibited a notable influence on the molecular structures, adsorption states, and bioactivity of BMP-2 [12,23]. However, the effects of zinc-substituted HAP (Zn-HAP) on its interactions with BMP-2 have not been clearly clarified until now, though Zn^2+^ is one of the commonly applied cation ions and a significant trace element in natural bone [24,25]. Thus, this work used hybrid MD and SMD simulations to investigate the adsorption and desorption processes of BMP-2 upon Zn-HAP surfaces (Zn-HAPs); meanwhile, experimental studies were performed to validate the theoretical findings. The substitution of Zn to Ca in Zn-HAP was designed at a series of molecule ratios, namely, 1 at%, 2.5 at%, 5 at%, and 10 at%. The interaction energy, adsorption and desorption states, molecule conformation, and secondary structures were deeply investigated in this work. The bioactivity of BMP-2 upon Zn-HAPs was discussed for the aspect of stability of cysteine-knots and changes in secondary structures; furthermore, it was confirmed by an alkaline phosphatase (ALP) activity study and a reverse transcription-polymerase chain reaction (RT-PCR) assay. The findings here could broaden the understanding of interactions between proteins and inorganic material surfaces, and provide useful guidelines for the experimental design of BMP-2-immobilized biomaterials.

## 2. Results

### 2.1. Interaction Energy

**Figure 1:**
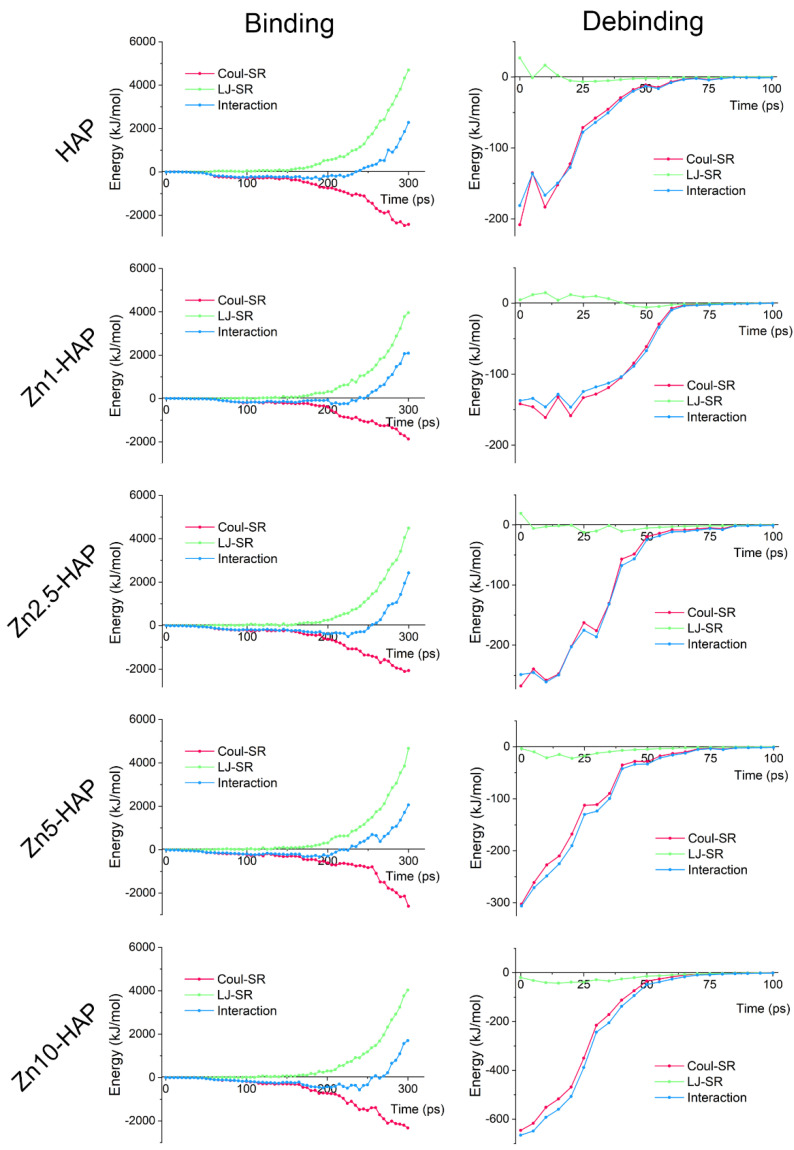
LJ-SR, Coul-SR, and interaction energy between BMP-2 and HAP/Zn-HAPs during the binding and debinding processes. The interaction energy is composed of LJ-SR (repulsive energy) and Coul-SR (attractive energy).

The LJ-SR, Coul-SR, and interaction energy during the binding and debinding processes are depicted in Figure 1. In the binding process, it can be found that LJ-SR (as repulsive energy) was almost zero at first, and rapidly increased after the critical distance between BMP-2 and Zn-HAPs. The Coul-SR (as attractive energy) was gradually decreased during BMP-2 approaching Zn-HAPs. The interaction energy, composed of LJ-SR and Coul-SR, can be divided into three stages in the binding process. Stage 1: it kept at nearly zero as LJ-SR and Coul-SR were all close to zero. Stage 2: the interaction energy slightly decreased and attained a peak value, which indicates BMP-2 preferred to adsorb upon the surface. Stage 3: the interaction energy increased rapidly due to the sharply amplified LJ-SR, which suggests BMP-2 tended to desorb from the surface. The binding energies (peak value of interaction energy, averaged over the adjacent 20 ps configurations) of BMP-2 were significantly different among the HAP and Zn-HAPs, as shown in Table 1. In detail, the binding energies of the Zn2.5-HAP and Zn10-HAP groups were notably higher (*p* < 0.05) than those of the other groups, which indicates that BMP-2 had a high adsorption affinity on the Zn2.5-HAP and Zn10-HAP surfaces. Moreover, the COM distance, SMD time, and number of contacts (NOC) revealed a similar trend to the results of binding energy (Table 1). These findings suggest that BMP-2 molecules, at the binding state, were nearer to the surfaces and had more contact points on the Zn2.5-HAP and Zn10-HAP surfaces than those on the HAP, Zn1-HAP, and Zn5-HAP surfaces.

In the debinding process, it can be found that LJ-SR was close to zero, while Coul-SR showed a considerable value below zero at first, and it rapidly increased near zero in the following. As a result, the interaction energy during the debinding process was mostly dominated by Coul-SR, which acted as attractive energy and tended to adsorb on the surface. At the beginning of the debinding process, LJ-SRs of the Zn1-HAP, Zn5-HAP, and Zn10-HAP groups were approximately equal to zero. This phenomenon was ascribed to the MD relaxation procedure induced rearrangement of BMP-2 and largely reduced the repulsive energy (LJ-SR). In addition, the HAP group revealed less debinding energy (interaction energy at time = 0 ps) than the Zn-HAPs groups, except for the Zn1-HAP group. Moreover, the Zn10-HAP group showed the highest debinding energy among all the groups. These results suggest that BMP-2 could be easily desorbed from HAP as compared to Zn-HAPs. More importantly, the desorption of BMP-2 was more difficult upon Zn-HAPs with high-amount substitution ratios of Zn/Ca than those with low amounts.

### 2.2. Structure States of BMP-2

**Figure 2:**
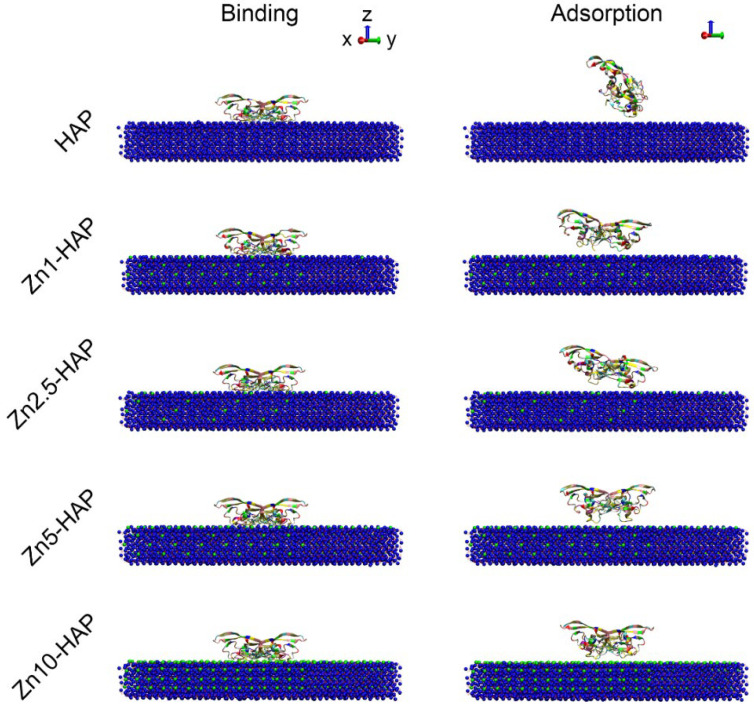
Snapshots of BMP-2 upon HAP and Zn-HAPs at the binding state and adsorption state. The conformations of BMP-2 at the binding state were similar to its initial state. However, the conformations of BMP-2 at the adsorption state were shifted significantly, especially for BMP-2 upon HAP.

While BMP-2 interacted with HAP/Zn-HAPs, the binding state and adsorption state of BMP-2 were obtained (as shown in Figure 2). It can be found that the conformations of BMP-2 at the binding state were not significantly different among all the groups. However, after the MD relaxation procedure, the conformations of BMP-2 at the adsorption state were obviously different among all the samples. For example, the BMP-2 molecule rotated nearly 90 degrees and achieved an “end-on” orientation on the HAP surface. The BMP-2 molecules of the Zn1-HAP and Zn2.5-HAP groups rotated slightly in the final adsorption state. The BMP-2 proteins did not rotate and achieved a “side-on” orientation on the Zn5-HAP and Zn10-HAP surfaces. In addition, we calculated the contact area between BMP-2 and HAP/Zn-HAPs by solvent-accessible surface area. As shown in Figure S1 (Supplementary Materials), the contact area rapidly increased in the SMD procedure, and the increased rates among the groups were not significantly different. However, the values of the contact area at the binding state (peak value of contact area) were ranked in the following order: HAP ≈ Zn5-HAP < Zn1-HAP < Zn2.5-HAP < Zn10-HAP. The contact area quickly decreased in the MD relaxation procedure due to the rearrangement of proteins in the adsorption process. The values of the contact area at the adsorption site were ranked in the following order: HAP ≈ Zn1-HAP < Zn2.5-HAP ≈ Zn5-HAP < Zn10-HAP. These results indicate that different substitution ratios of Zn/Ca notably influenced the contact area of BMP-2 on Zn-HAPs, and further mediated the molecular structures of BMP-2. However, the tendency of the effects of the Zn/Ca substitution amount on the contact area of BMP-2/Zn-HAPs was complex and elusive, which should be investigated in a further study.

**Figure 3:**
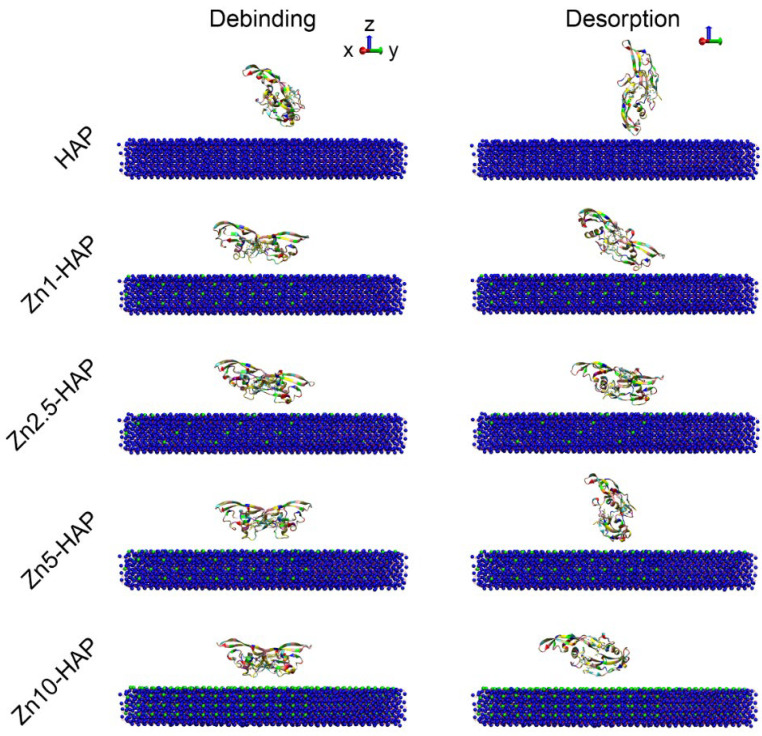
Snapshots of BMP-2 upon HAP and Zn-HAPs at the debinding state and desorption state. The conformations of BMP-2 at the debinding state were comparable to their adsorption states. However, the conformations of BMP-2 at the desorption states were remarkably changed.

In addition, the debinding and desorption states of BMP-2 upon HAP and Zn-HAPs are depicted in Figure 3. It can be found that the conformations of BMP-2 at the debinding state were not significantly different as compared to those at the adsorption state, but the distance between the protein and surface models was enlarged. The conformations of BMP-2 at the desorption states were remarkably changed when compared to their debinding states. For instance, the BMP-2 molecules of the HAP, Zn1-HAP, and Zn2.5-HAP groups were further rotated to some extent, and anchored to the surface in the desorption state. The BMP-2 protein of the Zn5-HAP group rotated to a huge extent, and achieved an “end-on” orientation upon Zn5-HAP. However, the BMP-2 protein of the Zn10-HAP group also significantly rotated and achieved a “lay-on” orientation upon the Zn10-HAP surface. The minimum distances between BMP-2 and surface at the desorption state were 0.254 ± 0.004, 0.253 ± 0.010, 0.554 ± 0.09, 0.738 ± 0.148, and 0.669 ± 0.072 nm for the HAP, Zn1-HAP, Zn2.5-HAP, Zn5-HAP, and Zn10-HAP groups, respectively. These findings suggest that even though BMP-2 detached from HAP and Zn-HAPs, it still could closely interact with the surface by one monomer of the protein (especially for HAP and Zn1-HAP). The contact area of BMP-2 upon HAP and Zn-HAPs during the debinding process was recorded in Figure S2 (Supplementary Materials). It clearly depicted that the values of the contact area were quickly decreased in the debinding process, and they attained nearly zero at the time of 80 ps of the reverse SMD procedure. These data confirmed that the BMP-2 molecules were separated from HAP and Zn-HAPs in the debinding process. Moreover, the values of the contact area at the first half of the debinding process were ranked in the following order: HAP ≈ Zn1-HAP < Zn2.5-HAP ≈ Zn5-HAP < Zn10-HAP, corresponding well with the contact area data at the adsorption sate.

### 2.3. Rg and RMSD

**Figure 4:**
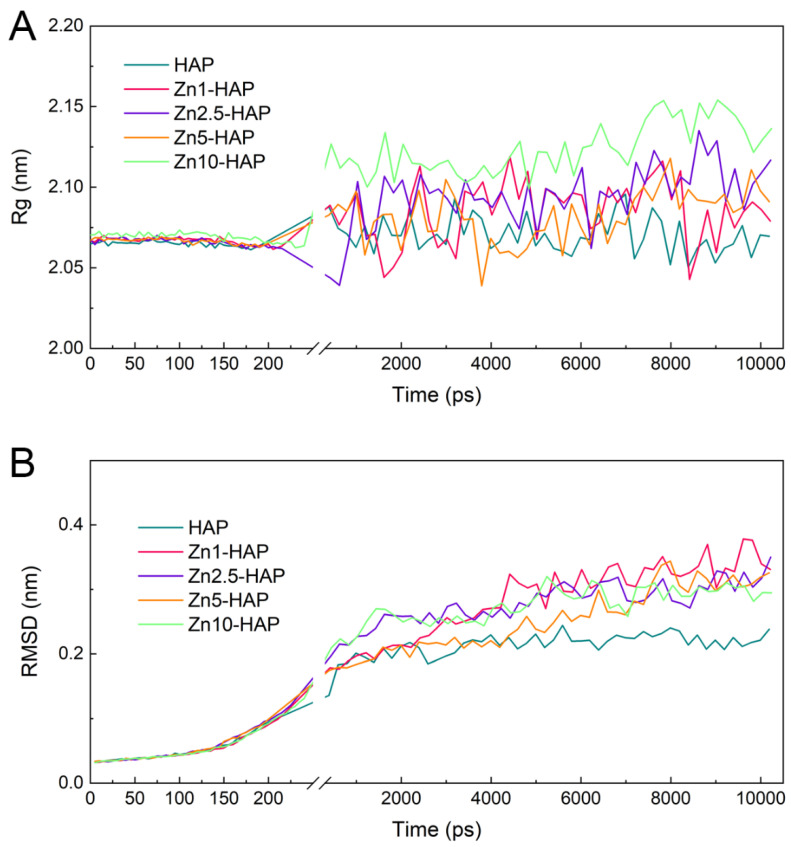
Rg (**A**) and RMSD (**B**) of BMP-2 upon HAP/Zn-HAPs during the binding and adsorption processes.

The molecular structures of BMP-2 were investigated during the adsorption and desorption processes. As shown in Figure 4A, the values of Rg of BMP-2 among all the groups were similar and kept nearly unchanged in the SMD procedure. This finding indicates that there was no serious compression in the BMP-2 molecule during the binding process. Moreover, the values of Rg of BMP-2 in the MD relaxation procedure were slightly changed among the HAP and Zn-HAP samples. It should be mentioned that the value of Rg of BMP-2 upon HAP at the adsorption state was the lowest, while that of the Zn10-HAP group was the highest, suggesting a compact structure of BMP-2 on HAP and a loosened structure on Zn10-HAP. The results of RMSD were corresponding well with the results of Rg. As shown in Figure 4B, the values of RMSD of BMP-2 among all the groups were comparable. They all slightly increased at the first 120 ps of SMD procedure and quickly increased in the following ~100 ps of SMD procedure. The values of RMSD of BMP-2 in the MD relaxation procedure were slightly shifted for Zn-HAPs and even kept unchanged for HAP, demonstrating that BMP-2 achieved a stable state upon these surfaces.

We also investigated the Rg and RMSD in the debinding and desorption processes. As shown in Figure S3A (Supplementary Materials), the values of Rg of BMP-2 among all the groups remained stable in the debinding process, and they were obviously increased in the following order: HAP < Zn1-HAP < Zn5-HAP < Zn2.5-HAP < Zn10-HAP. In the desorption (MD relaxation) process, the values of Rg of BMP-2 were shifted to some extent but were still comparable to those of the debinding process. The data of RMSD of BMP-2 showed a similar trend to the findings of Rg in the debinding and desorption processes (Figure S3B, Supplementary Materials). Moreover, the values of RMSD of BMP-2 were sharply amplified in the first 100 ps of the desorption (MD relaxation) process, which was ascribed to the disappeared short-term BMP-2/Zn-HAP interactions. These findings suggest that substitution of Zn/Ca, especially with a high molecule ratio, in the HAP surface, could enhance the structural rearrangement of BMP-2 and achieve a loosened configuration of BMP-2 in the adsorption and desorption processes.

### 2.4. RMSF and R_f_

**Figure 5:**
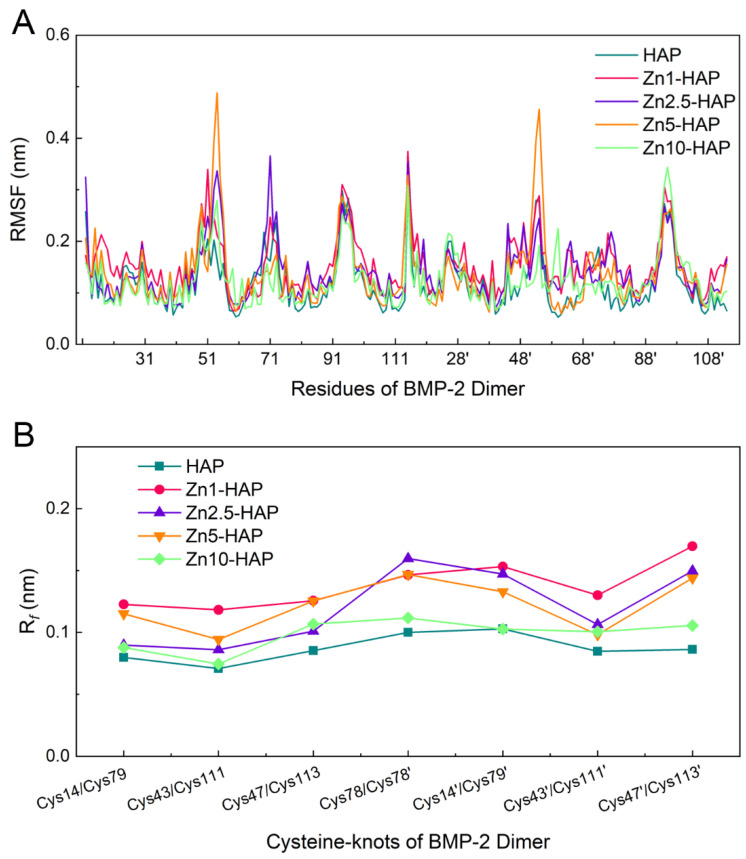
RMSF (**A**) of BMP-2 residues and R*_f_* (**B**) of cystine-knots of BMP-2 upon HAP and Zn-HAPs during the adsorption (MD relaxation) process.

To investigate the flexibility of BMP-2 upon HAP and Zn-HAPs, we measured the RMSF of BMP-2 residues during the adsorption process (as shown in Figure 5A). It can be found that the values of RMSF of the majority residues were approximately 0.1 nm, while those of several residues were relatively high (~0.3 nm). In detail, the most flexible residues of BMP-2 dimer were from ALA52 to ASN56, from VAL70 to LYS73, from ASP93 to LYS97, from SER12′ to CYS14′, from ALA52′ to LEU55′, and from GLU94′ to LYS97′, which suggests that the corresponding residues had relatively higher flexibility. The values of RMSF of BMP-2 residues between the HAP and Zn-HAPs groups were similar. However, the RMSF values of ALA52, ASP53, HIS54, LEU55, and ASN56 residues in both monomers of the Zn5-HAP group were notably higher than those of the other groups. This finding indicates that BMP-2 on the Zn5-HAP surface may lose its biological activity due to its too high flexibility. In addition, the R*_f_* (RMSF of cysteine-knots of BMP-2 dimer) was calculated and is depicted in Figure 5B. The values of R*_f_* of all groups were kept at nearly 0.1 nm, which indicates that the flexibility of cysteine-knots was comparable to the whole molecule. Moreover, the R*_f_* values of the HAP and Zn10-HAP groups were relatively low as compared to those of the Zn1-HAP, Zn2.5-HAP, and Zn5-HAP groups. Since cysteine-knots stabilize the structure and bioactivity of BMP-2 [12], the HAP and Zn10-HAP groups, which owned the low flexibility of cysteine-knots of BMP-2, could exhibit higher bioactivity of adsorbed BMP-2 than the other groups.

### 2.5. Secondary Structures

**Figure 6:**
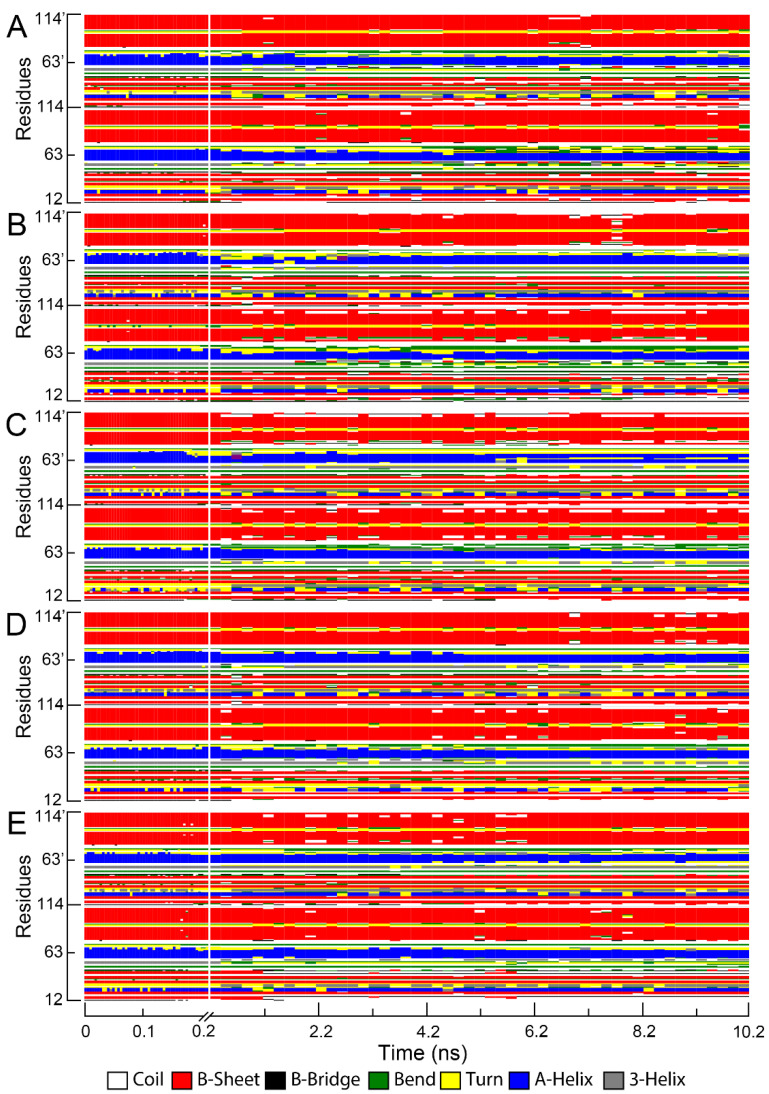
Secondary structures of BMP-2 upon HAP and Zn-HAPs during the binding and adsorption processes. (0 to ~0.2 ns: binding process; ~0.2 to ~10.2 ns: adsorption process. (**A**), HAP; (**B**), Zn1-HAP; (**C**), Zn2.5-HAP; (**D**), Zn5-HAP; (**E**), Zn10-HAP).

To further analyze the structure and bioactivity of BMP-2, the secondary structures of BMP-2 dimer during the adsorption and desorption processes were calculated (Figure 6 and Figure S4, Supplementary Materials). It was found that BMP-2 molecules maintained a large segment of β-sheet and α-helix structures during the adsorption and desorption processes. Moreover, the secondary structures of BMP-2 upon HAP and Zn-HAPs were not significantly changed. These results indicate that the main structures of BMP-2 were well-remained during the adsorption and desorption processes.

However, there were some discrepancies in the secondary structures of BMP-2 among the HAP and Zn-HAPs groups. As revealed in Table S2 (Supplementary Materials), the content of random coil structures of BMP-2 at the initial state (in solution) was 22.1 ± 1.3%. The contents of random coil structures of BMP-2 upon HAP at the adsorption and desorption states were 21.5 ± 1.7% and 20.7 ± 1.3%, respectively. However, the contents of random coil structures of BMP-2 upon Zn-HAPs at the adsorption and desorption states were all ~25% (Table S2, Supplementary Materials). These results demonstrate that when interacting with the HAP surface, BMP-2 tended to slightly decrease the random coil structures; but when interacting with the Zn-HAPs surfaces, BMP-2 tended to slightly increase the random coil structures. In addition, the contents of α-helix of BMP-2 on HAP and Zn-HAPs at the adsorption and desorption states were significantly decreased. In particular, the HAP and Zn1-HAP groups showed a ~3% decrease at the desorption state. The contents of the β-sheet of BMP-2 varied slightly upon HAP and Zn-HAPs at the adsorption and desorption states. For instance, the contents of the β-sheet of BMP-2 on Zn10-HAP at the adsorption and desorption states were 42.2 ± 1.2% and 38.2 ± 3.2%, respectively. Given that the content of the β-sheet of BMP-2 at the initial state was 42.3 ± 1.4%, the BMP-2 adsorbed on Zn10-HAP well-remained its β-sheet structures; however, the BMP-2 desorbed from Zn10-HAP significantly reduced its β-sheet structures. Moreover, slightly decreased contents of β-turn structures were found on the HAP and Zn-HAPs groups both at the adsorption and desorption states, as compared to 10.2 ± 1.6% of β-sheet of BMP-2 at the initial state. The contents of β-bend of BMP-2 upon HAP at the adsorption and desorption states significantly increased more than its initial state. Particularly, the HAP group exhibited a ~3% increase in β-bend of BMP-2 at the desorption state. However, the contents of β-bend of BMP-2 upon Zn-HAPs at the adsorption and desorption states were comparable to its initial state, except for Zn10-HAP, which showed a ~1% increase in β-bend of BMP-2 at the desorption state. Altogether, Zn-HAPs could remarkably influence the secondary structures of BMP-2 during its adsorption and desorption processes. It should be noted that the adsorption of BMP-2 on Zn10-HAP can benefit its bioactivity since the secondary structures were largely maintained, but the desorption process might disturb the bioactivity of BMP-2.

### 2.6. Osteogenic Activity

**Figure 7:**
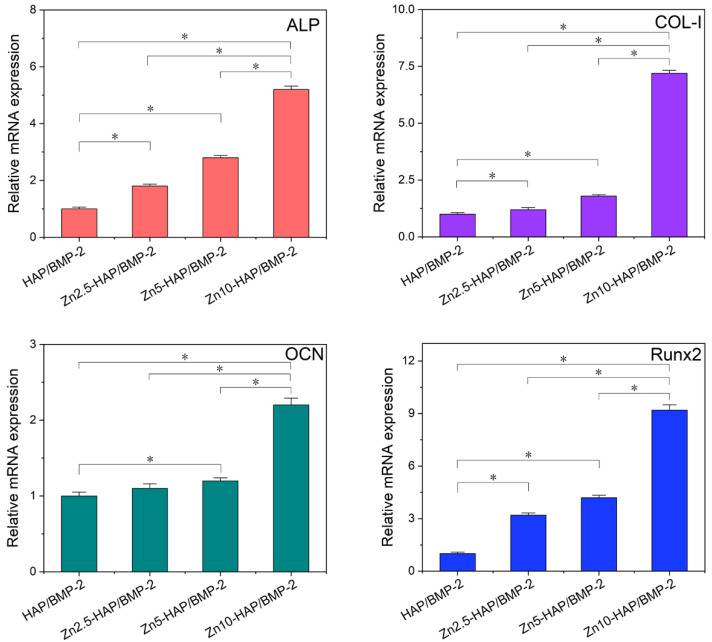
Relative osteogenic genes (ALP, COL-I, OCN, and Runx2) expression of C2C12 cells after incubation with HAP/BMP-2 and Zn-HAPs/BMP-2 for 3 days. Expression levels are normalized with total GAPDH content and the HAP/BMP-2 group. * *p* < 0.05, compared with corresponding samples.

To validate the conclusion of the theoretical simulation, ALP activity and osteogenic marker genes of C2C12 cells incubated with HAP/BMP-2 and Zn-HAPs/BMP-2 were measured in this work. The values of ALP activity of C2C12 cultured with Zn-HAPs/BMP-2 were significantly higher than that of the HAP/BMP-2 group (as indicated in Figure S5, Supplementary Materials). Importantly, Zn10-HAP/BMP-2 revealed the highest ALP activity as compared to Zn2.5-HAP/BMP-2 and Zn5-HAP/BMP-2. As shown in Figure 7, relative mRNA expressions of ALP, COL-I, OCN, and Runx2 showed a similar trend to the results of ALP activity. It can be inferred that, compared to pure HAP, the substitution (ratios < 10 at%) of Zn/Ca for Zn-HAPs could enhance the osteogenic activity of BMP-2 when adsorbed upon these surfaces. Importantly, the highest osteogenic bioactivity of BMP-2 was achieved on the Zn10-HAP group (with a 10 at% substitution ratio). Intriguingly, these experimental results correspond well with the conclusions of theoretical modeling, which propose that the bioactivity of BMP-2 upon Zn10-HAP can be well preserved or even enhanced in contrast to other surfaces.

## 3. Discussion

Zn, an essential trace element for the human body, is required for protein synthesis, DNA synthesis, cell mitosis, and cell proliferation. In the field of bone tissue engineering, it was reported that Zn could stimulate ALP activity, enhance bone mineralization, and inhibit bone resorption [26,27]. Therefore, Zn-contained biomaterials were commonly developed and utilized for accelerating bone regeneration. For example, an in vivo study reported that BMP-2-loaded Zn-HAP showed significantly higher new bone formation than the BMP-2-loaded HAP/collagen group and the bare control group [24]. However, the underlying mechanisms regarding how BMP-2/Zn-HAP interactions influence osteogenic activity at the molecule level and the corresponding cell studies are still missing. This work thoroughly investigated the interactions between BMP-2 and Zn-HAPs at the atom level. We found that Zn-HAPs (especially with a 10 at% substitution) benefited the immobilization of BMP-2 and, importantly, could better stabilize the bioactivity of BMP-2. These findings were further confirmed by the ALP study and RT-PCR assay, which showed that the osteogenic activity of BMP-2 was significantly enhanced upon Zn-HAPs. It should be mentioned that the BMP-2-adsorbed Zn-HAPs showed increased osteogenic bioactivity along with increasing Zn/Ca substitution ratios. The reason for this phenomenon is that Zn favors cell metabolism and stabilizes molecular structures of BMP-2. As a result, a 10 at% substitution of Zn/Ca exhibited the highest osteogenic activity of BMP-2 as compared to other samples.

It was found that the binding energies of BMP-2 upon Zn-HAPs were notably higher than that of HAP (Table 1). As a result, the adsorbed BMP-2 molecules may be closer to the Zn-HAPs surfaces but not to the HAP surface, which was demonstrated by the data of the COM distance. Meanwhile, the NOC and contact area were notably enhanced for the Zn-HAPs groups. These findings indicate that the binding affinity of BMP-2/Zn-HAPs was remarkably higher than that of BMP-2/HAP. It can be inferred that, compared to HAP, Zn-HAPs could load more BMP-2 molecules and thus could exhibit strong osteogenic capacity. In addition, the debinding energies of BMP-2 upon Zn-HAPs were significantly bigger than that of HAP (Figure 1). This finding signifies that the detachment of BMP-2 molecules from the Zn-HAPs surfaces was more difficult than that from the HAP surface, which could benefit the bioactivity of BMP-2. It is known that too strong an interaction capacity of BMP-2/biomaterials may interrupt the recognition of cellular receptors and affect the bioactivity of BMP-2 [5,28]. In the present study, the interaction energies between BMP-2 and HAP/Zn-HAPs were not too high as compared to those of related studies [19,20,29]. In addition, the interaction energies in this work are different as compared to other similar works [30,31]. This discrepancy is ascribed to the different BMP-2 protein ID (3BMP vs. 1REW) and force field parameters for HAP (Hauptmann et al.’s vs. INTERFACE force fields) utilized in these studies. Thus, bigger interaction energies for the BMP-2/Zn-HAPs systems could benefit the loading capacity of BMP-2. Therefore, Zn10-HAP, which revealed the high binding energy of BMP-2, should own both high loading efficiency and good bioactivity.

Intriguingly, molecular structures of BMP-2 were well stabilized upon Zn-HAPs in comparison to HAP. This conclusion could be explained by (i) snapshots of BMP-2 upon HAP and Zn-HAPs and (ii) changes in the secondary structures of BMP-2. The snapshots at the adsorption state showed that the BMP-2 molecule rotated to some extent upon the HAP surface (Figure 2). Moreover, some molecular structure disruption could be seen in the HAP group. However, BMP-2 molecules maintained the majority of structures upon the Zn-HAPs surfaces. The snapshots at the desorption state revealed that the conformation of the BMP-2 molecule remarkably shifted upon both HAP and Zn-HAPs as compared to those at the debinding state (Figure 3). Large conformation changes in BMP-2 at the desorption state should affect its bioactivity from the viewpoint of MD simulation. By using DSSP, we found that secondary structures of BMP-2 changed to some extent upon both HAP and Zn-HAPs (Table S2, Supplementary Materials) as compared to its initial state. The changes in the secondary structures were slightly bigger for the HAP group than those of the Zn-HAPs groups. For example, ~1.8% and ~3.4% decreases in α-helix were found on HAP at the adsorption and desorption states, respectively. In contrast, an average of ~1.6% and ~2.1% decreases in α-helix were reported on Zn-HAPs at the adsorption and desorption states, respectively. Taken together, compared to pure HAP, Zn-HAPs could better maintain the secondary structures of BMP-2, and thus could well preserve the bioactivity of BMP-2.

It should be noted that different substitution amounts of Zn-HAPs also exhibited significant influences on the adsorption and desorption of BMP-2. There is a non-linear correlation between the substitution ratios of Zn/Ca and the binding energies of BMP-2/Zn-HAPs (Table 1). A positive correlation between the substitution ratios of Zn/Ca and the debinding energies of BMP-2/Zn-HAPs can be found. These findings indicate that the influence of substitution amounts on the BMP-2/substrate interactions was complex. It is found that Zn1-HAP, Zn2.5-HAP, and Zn5-HAP decreased the stability of cystine-knots of BMP-2 at the adsorption process (Figure 5). However, Zn10-HAP well preserved the stability of cystine-knots of BMP-2 at the adsorption process, which should achieve high biological activity of BMP-2 [12]. In addition, our cellular experimental studies confirmed that Zn-HAPs/BMP-2 (especially Zn10-HAP/BMP-2) revealed higher osteogenic bioactivity than HAP/MP-2. Previous studies of cations-substituted HAP (e.g., Mg^2+^, Sr^2+^) revealed that an appropriate amount of substitution (e.g., 5–10 wt% of Sr/calcium phosphate cement) significantly enhanced the bioactivity of BMP-2 [23]. Corresponding well with these reports, we propose that a 10 at% substitution of Zn/Ca could offer high bioactivity of adsorbed BMP-2.

## 4. Materials and Methods

### 4.1. Protein Model

The initial molecular structure of BMP-2 was adopted from Protein Data Bank (ID = 1REW, dimer with its type IA receptor, 1.86 Å resolution). The receptor molecules and crystallographic water molecules were discarded. It should be noted that the first 12 residues of each BMP-2 monomer were missing; thus, the starting protein model was composed of two sequences of 103 residues (starting from 13 and ending at 114) [20]. Considering that our previous works [12,30] investigated various orientations of BMP-2 upon biomaterials, the present study used one of the most popular orientations (Side1) and concentrated on the influences of molecule ratios of Zn/Ca on the adsorption and desorption of BMP-2 upon Zn-HAPs.

### 4.2. Surface Models

The single unit cell of HAP (space group P6_3_/m, *a* = *b* = 0.943 nm, *c* = 0.688 nm) was taken from American Mineralogist Crystal Structure Database. HAP surface model ((0 0 1) face, 15 × 15 × 4 super cells) was constructed by using Materials Studio software (Accelrys Software Inc., San Diego, CA, USA). To develop Zn-HAPs, uniform substitutions of Ca^2+^ by Zn^2+^ at different mole fractions (1 at%, 2.5 at%, 5 at%, and 10 at%) were performed. It should be mentioned that the substitution took place at the upper surface of each single unit cell; thus, it could benefit the assessment of Zn-influenced adsorption and desorption of BMP-2.

### 4.3. System Setup

The computer simulations were performed using the latest GROMACS software. It is known that the OPLS-AA force field was widely applied in MD simulations of polypeptide, protein, nucleic acid, organic solvent, and other liquid systems. Recently, the INTERFACE force field was developed for some inorganic compounds (including HAP), to enable accurate simulations of inorganic/organic and inorganic/biomolecular interfaces [28,32]. Thus, accurate force field parameters of HAP from the INTERFACE force field were integrated into the OPLS-AA force field in this work to tackle the interactions between Zn-HAPs and the BMP-2 molecule. The BMP-2 and Zn-HAP complexes were put into a system box (24.5 × 15 × 12 nm), and set center as 12.25 × 7.5 × 3 nm to fully hold the complexes as well as to save system volume. Single point charge (SPC) water molecules were utilized as a solvent in the system box, and further randomly substituted by a few calcium and chloride ions to neutralize the system. All simulations were performed under periodic boundary conditions with a 2 fs time step. The particle mesh Ewald algorithm was employed to calculate the long-range electrostatic interactions. The electrostatic cut-off distance and van der Waals cut-off distance were both set as 10 Å.

### 4.4. Computer Simulations

An energy minimization procedure was performed on the BMP-2/Zn-HAP system, and stopped when the maximum force between all atoms was less than 1000 kJ mol^−1^ nm^−1^. Subsequently, a 50 ps NVT ensemble (T = 300 K) and a 50 ps NPT ensemble (P = 1 bar, T = 300 K) were performed, respectively, in order to achieve thermal equilibration for the systems. Following this, a four-step protocol of combined SMD and MD simulations was carried out. Step 1: binding process, 300 ps SMD procedure with *constant velocity pulling* (PCV, *k* = 1000 kJ mol^−1^ nm^−2^, *v* = 0.005 nm ps^−1^) was conducted to simulate the early stage of the adsorption process. The coordinates of the systems at the binding state were investigated by the binding energy and utilized in the following steps. Step 2: adsorption process, 10 ns MD relaxation procedure was performed to imitate the rearrangement of protein adsorption. Step 3: debinding process, 100 ps reverse SMD procedure with *constant velocity pushing* (P’CV, *k* = 1000 kJ mol^−1^ nm^−2^, *v* = −0.005 nm ps^−1^) was conducted to simulate the early stage of the desorption process. Step 4: desorption process, 10 ns MD relaxation procedure was performed to imitate the rebalance of protein desorption.

### 4.5. Data Collection

The short-range Lennard-Jones energies (LJ-SR, van der Waals interaction) and Coulombic potentials within R-coulomb (Coul-SR, electrostatic interaction) were recorded to study the interaction energy during the binding and debinding processes. Meanwhile, the center of mass (COM) distance, SMD time, and number of contacts (NOC) at the binding state were collected. The contact area, radius of gyration (Rg), root mean square deviation (RMSD), and root mean square fluctuation (RMSF) were also obtained. Moreover, the RMSF of cysteine-knots of BMP-2 (R*_f_*, stability index of cysteine-knots) was calculated according to our previous studies [12,31]. The secondary structures of BMP-2 were analyzed using a program of DSSP [30]. In addition, the contents of secondary structures of BMP-2 at the initial, binding, adsorption, debinding, and desorption states were calculated.

### 4.6. Experimental Studies

To validate the bioactivity of BMP-2 adsorbed upon HAP and Zn-HAPs, experimental studies including ALP study and RT-PCR assay were performed. The preparation process of HAP and Zn-HAP coatings was as follows: HAP and Zn-HAP (1 at%, 2.5 at%, 5 at%, and 10 at%) nanoparticles were fabricated by a microwave method according to our previous study [12]. The prepared HAP and Zn-HAP nanoparticles were mixed into a paste with curing liquid in a liquid-to-solid ratio of 250 L/g, and packed in a stainless-steel mold (10 × 2 mm, diameter × height) at 2 MPa for 1 min, after which the resulted samples were placed at 37 °C and 100% relative humidity for 72 h. The obtained HAP and Zn-HAP compacts exhibited no serious disintegration and only released a few nanoparticles in the cell culture medium, and thus did not notably influence cellular studies.

#### 4.6.1. ALP Study

We measured the ALP activity of C2C12 cells cultured with BMP-2-adsorbed HAP and Zn-HAPs. In brief, 0.2 mL of C2C12 cells (5 × 10^4^ cells mL^−1^) was seeded upon the samples (in a 24-well plate) and cultured for 3 days. Then, samples were incubated with a lysis buffer (1% NP-40 in PBS) for 90 min, and further centrifugated at 800 rpm for 5 min to obtain cell lysate. Then, 50 μL of the obtained cell lysate was mixed with 100 μL of PNPP-Na (2.5 mg/mL) solution and incubated for 30 min. After which, 0.1 mL of 0.1 M NaOH was added to the mixed solution to stop the reaction. The OD value was quantified at a wavelength of 405 nm using a microplate reader (SPECTRAmax 384, Molecular Devices, San Jose, CA, USA). The total amount of protein in each sample was measured by a BCA assay kit (Beyotime, Shanghai, China). The ALP activity was normalized to the total protein content and the reaction time (OD/mg protein/min).

#### 4.6.2. RT-PCR Assay

The effects of Zn-HAPs and HAP on the osteogenic bioactivity of BMP-2 were further explored by quantitative RT-PCR analysis. All RT-PCR procedures were performed according to the previous works [7,23]. Briefly, 1 mL of C2C12 cells (1 × 10^5^ cells mL^−1^) was incubated with the Zn-HAPs/BMP-2 and HAP/BMP-2 samples (in a 6-well plate) for 3 days. Possible spalled-off nanoparticles from HAP and Zn-HAP compacts were separated by centrifugation (800 rpm, 5 min). The obtained C2C12 cells were incubated with Trizol Reagent Kit (Takara, Kusatsu, Shiga, Japan) to extract total RNA. The RNA samples were converted into cDNA by using the PrimeScript Reverse Transcription Reagent Kit (Takara, Kusatsu, Shiga, Japan). Subsequently, the quantification of all cDNA was performed by using the real-time PCR system (Bio-Rad, Hercules, CA, USA) with SYBR Premix Ex Taq Reagent Kit (Takara, Kusatsu, Shiga, Japan). The primer pairs of osteogenic marker genes (ALP, COL-I, OCN, Runx2, and GAPDH, Shenggong, Shanghai, China) are shown in Table S1 (Supplementary Materials). The relative mRNA expressions were normalized to HAP/BMP-2 and GAPDH.

### 4.7. Statistical Analysis

Statistical analysis was performed by using a one-way analysis of variance (ANOVA) with a Newman–Keuls multiple comparisons post hoc test. Statistically significant difference was considered with a *p* value less than 0.05.

## 5. Conclusions

In closing, this work employed a BMP-2 dimer to investigate its interactions with Zn-HAPs. The influence of different substitution ratios of Zn/Ca on the molecular structure, binding affinity, and biological function was deeply discussed through theoretical modeling and experimental study. It is demonstrated that BMP-2 revealed a high binding affinity upon Zn-HAPs as compared to pure HAP. Meanwhile, the adsorbed BMP-2 molecule could not be easily detached from the Zn-HAPs materials. In addition, the molecular structures of BMP-2 could be well stabilized by Zn-HAPs. Particularly, Zn10-HAP significantly preserved the stability of cystine-knots of BMP-2, and could benefit the bioactivity of BMP-2. The present study verified that Zn-HAPs materials could leverage the adsorption and bioactivity of BMP-2. The promising findings in this work could provide useful ideas for the design of BMP-2-involved new biomaterials for bone tissue engineering.

## Figures and Tables

**Table 1:** Interaction energy (kJ/mol), center of mass (COM) distance (nm), SMD time (ps), and number of contacts (NOC, <0.3 nm, count by residues) of BMP-2 upon HAP and Zn-HAPs at the binding state.

Surfaces	Interaction Energy	COM Distance	SMD Time	NOC
HAP	−257 ± 60	2.86 ± 0.04	190 ± 10	48.2 ± 10.1
Zn1-HAP	−236 ± 23	2.75 ± 0.04	215 ± 10	68.4 ± 8.2
Zn2.5-HAP	−383 ± 76	2.68 ± 0.04	225 ± 10	83.2 ± 9.9
Zn5-HAP	−291 ± 36	2.86 ± 0.04	190 ± 10	38.2 ± 4.7
Zn10-HAP	−397 ± 98	2.58± 0.04	240 ± 10	105.6 ± 5.6

## Data Availability

The data presented in this study are available on request from the corresponding authors with proper reasons.

## Supplementary Materials

The supporting information can be downloaded at: https://www.mdpi.com/article/10.3390/ijms231710144/s1.

## Abbreviations

BMPs	bone morphogenetic proteins
BMP-2	bone morphogenetic protein-2
Zn-HAP	zinc-substituted hydroxyapatite
MD	molecular dynamics
SMD	steered molecular dynamics
SPC	single point charge
PCV	constant velocity pulling
P’CV	constant velocity pushing
LJ-SR	short-range Lennard-Jones energies
Coul-SR	Coulombic potentials within R-coulomb
Rg	radius of gyration
RMSD	root mean square deviation
RMSF	root mean square fluctuation
R*_f_*	RMSF of cysteine-knots
COM	center of mass
NOC	number of contacts
ALP	alkaline phosphatase
RT-PCR	reverse transcription-polymerase chain reaction
