# Supplementary material for: Effects of Substitution Ratios of Zinc-Substituted Hydroxyapatite on Adsorption and Desorption Behaviors of Bone Morphogenetic Protein-2
Source: Int J Mol Sci. 2022 Sep 4;23(17):10144. doi: 10.3390/ijms231710144 (PMC9456158; doi:10.3390/ijms231710144)
Supplement: Supplementary file 1 [file ijms-23-10144-s001.zip › ijms-1852901-supplementary.pdf]

Table S1 The primer pairs of osteogenic gene ALP, COL-I, OCN, Runx2, and GAPDH.

Gene	Direction	Sequence (5’ - 3’)
ALP	Forward	CCAACTCTTTTGTGCCAGAGA
	Reverse	GGCTACATTGGTGTTGAGCTTTT
COL-I	Forward	GGTATGCTTGATCTGTATCTGC
	Reverse	AGTCCAGTTCTTCATTGCATT
OCN	Forward	CTGACAAAGCCTTCATGTCCAA
	Reverse	GCGGGCGAGTCTGTTCACTA
Runx2	Forward	CGGCCCTCCCTGAACTCT
	Reverse	TGCCTGCCTGGGATCTGT
GAPDH	Forward	GTCGTGGAGTCTACTGGTGTC
	Reverse	GAGCCCTTCCACAATGCCAAA

Table S2. Secondary structures of BMP-2 at the initial, binding, adsorption, debinding, and desorption states on HAP and Zn-HAPs.

		α-helix (%)	β-sheet (%)	β-turn (%)	β-bend (%)	Random coil (%)
Initial secondary structure		13.3 ± 1.4	42.3 ± 1.4	10.2 ± 1.6	7.6 ± 1.3	22.1 ± 1.3
HAP	Binding	14.6 ± 1.5	43.7 ± 0.1	8.2 ± 1.3	6.0 ± 0.8	22.8 ± 0.6
	Adsorption	11.5 ± 0.9	44.4 ± 2.2	9.9 ± 2.1	8.3 ± 1.2	21.5 ± 1.7
	Debinding	11.9 ± 0.3	43.7 ± 1.7	8.0 ± 1.9	9.6 ± 1.7	21.5 ± 1.3
	Desorption	9.9 ± 1.6	45.0 ± 1.4	8.4 ± 1.3	10.7 ± 1.8	20.7 ± 1.3
Zn1-HAP	Binding	12.7 ± 0.9	43.1 ± 0.8	8.9 ± 0.5	7.7 ± 0.8	21.8 ± 1.0
	Adsorption	12.1 ± 1.0	40.1 ± 2.4	8.1 ± 1.0	8.3 ± 1.0	25.0 ± 1.7
	Debinding	11.2 ± 1.4	43.9 ± 1.6	8.4 ± 1.6	6.6 ± 0.7	22.4 ± 1.1
	Desorption	10.5 ± 1.9	40.5 ± 3.9	9.3 ± 1.4	6.9 ± 1.1	25.3 ± 2.5
Zn2.5-HAP	Binding	10.8 ± 0.9	43.5 ± 0.4	10.8 ± 1.5	7.5 ± 0.4	23.6 ± 0.9
	Adsorption	11.1 ± 1.7	41.6 ± 2.0	10.0 ± 1.5	7.3 ± 1.3	25.3 ± 1.7
	Debinding	11.7 ± 0.8	43.5 ± 0.8	9.3 ± 0.2	6.8 ± 0.5	24.1 ± 0.6
	Desorption	10.7 ± 1.1	41.6 ± 1.6	9.5 ± 1.6	6.4 ± 0.7	25.9 ± 1.6
Zn5-HAP	Binding	15.5 ± 1.0	43.4 ± 0.7	7.2 ± 1.3	5.9 ± 0.2	22.5 ± 0.7
	Adsorption	11.8 ± 0.7	41.7 ± 1.8	9.8 ± 1.7	7.8 ± 0.6	24.8 ± 1.8
	Debinding	11.8 ± 0.9	42.5 ± 0.4	11.7 ± 1.2	7.8 ± 0.6	24.1 ± 0.3
	Desorption	12.1 ± 2.0	42.9 ± 2.9	9.1 ± 2.2	6.6 ± 1.3	23.4 ± 2.0
Zn10-HAP	Binding	12.2 ± 0.4	43.4 ± 0.7	8.6 ± 0.4	7.5 ± 0.3	24.6 ± 0.7
	Adsorption	11.7 ± 0.8	42.2 ± 1.2	9.4 ± 0.8	7.0 ± 1.0	23.9 ± 1.2
	Debinding	12.3 ± 0.4	41.1 ± 1.2	10.1 ± 0.9	6.7 ± 1.1	24.1 ± 1.2
	Desorption	11.5 ± 1.0	38.2 ± 3.2	8.8 ± 1.8	8.5 ± 1.2	26.2 ± 2.5

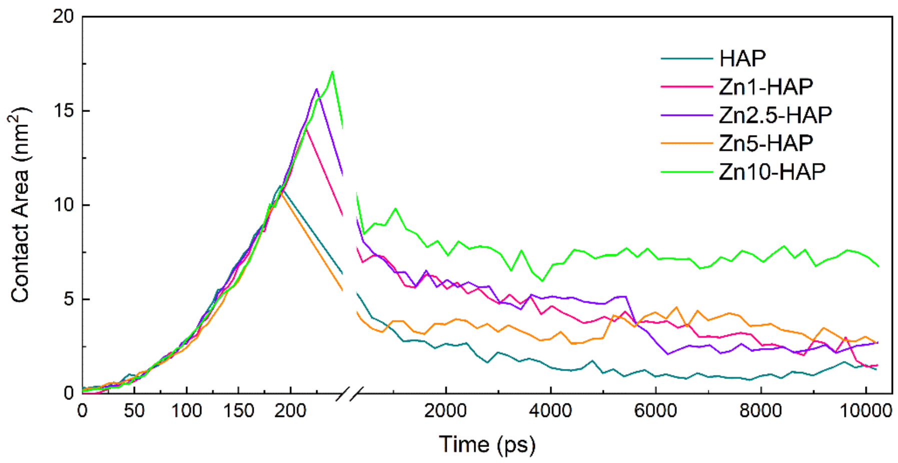

Figure S1. Contact area of BMP-2 upon HAP/Zn-HAPs during the binding and adsorption processes.

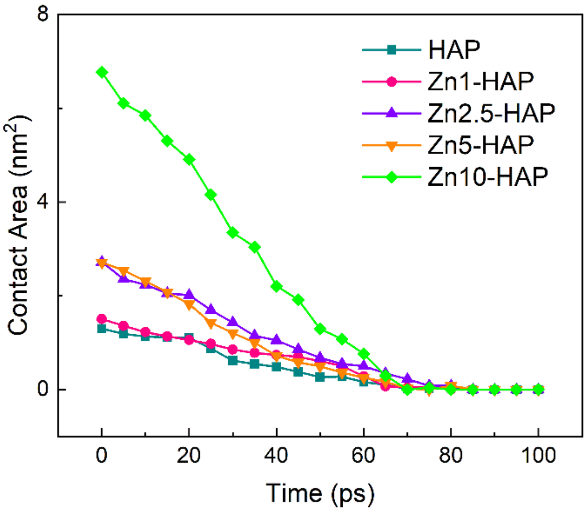

Figure S2. Contact area of BMP-2 upon HAP/Zn-HAPs during the debinding process.

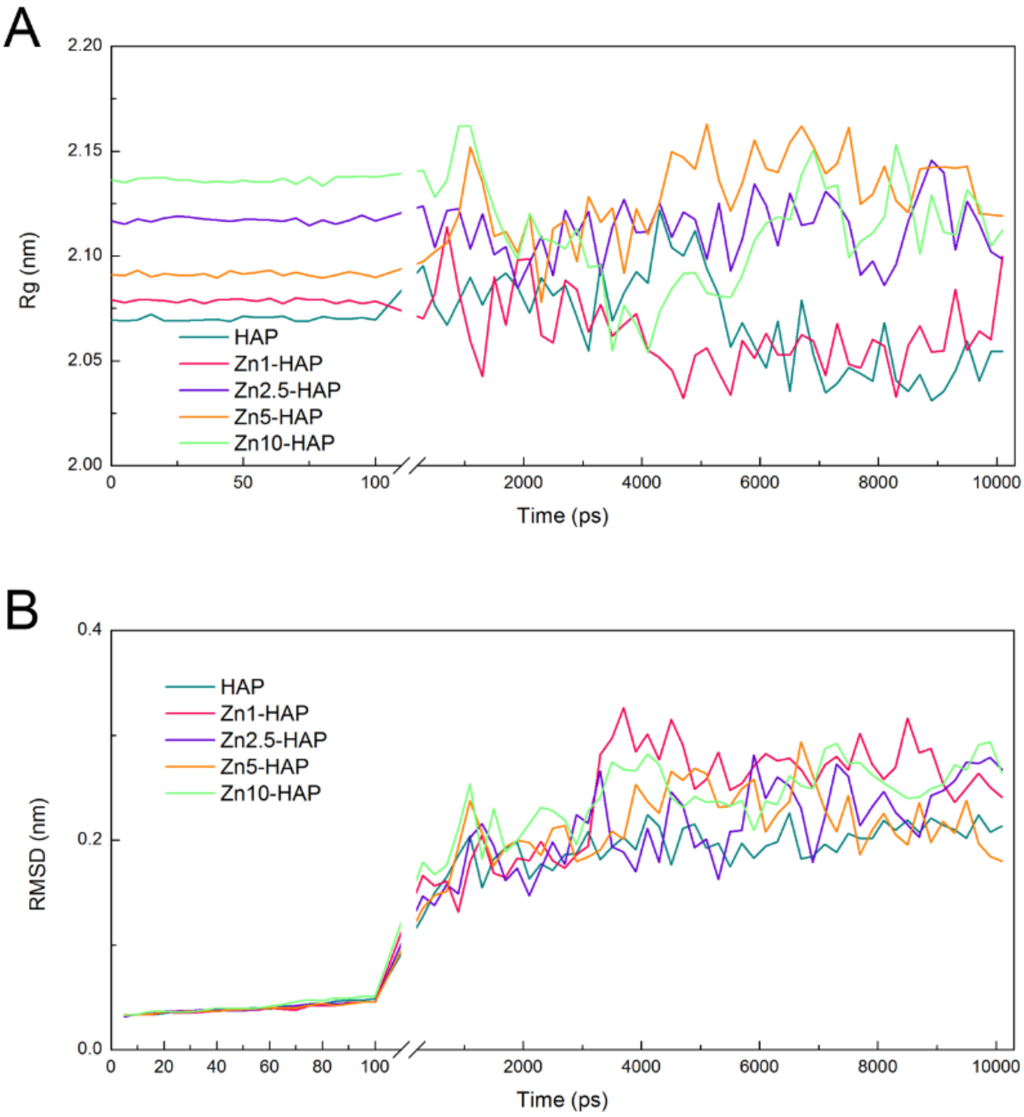

Figure S3. Rg (A) and RMSD (B) of BMP-2 upon HAP/Zn-HAPs during the debinding and desorption processes.

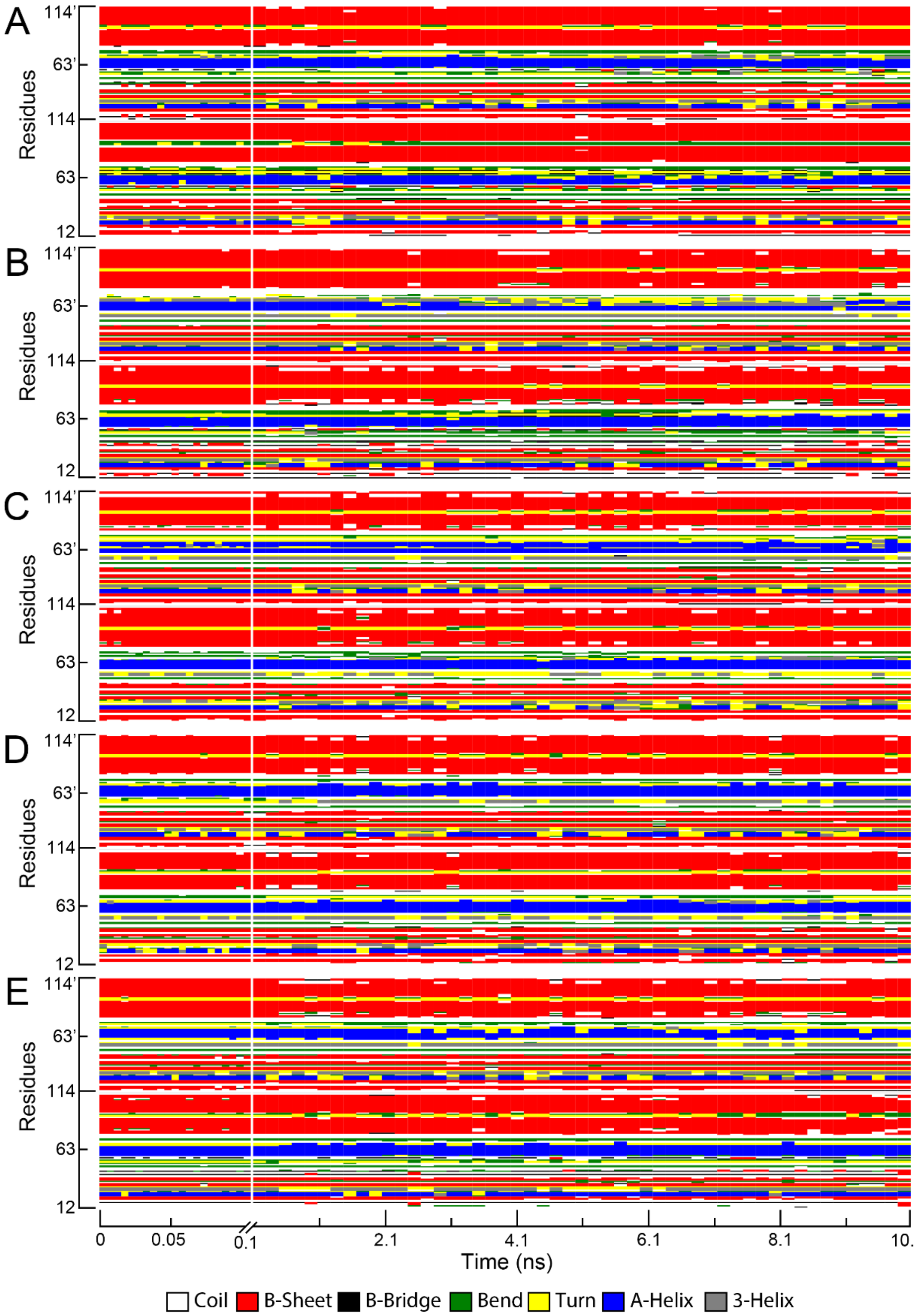

Figure S4. Secondary structures of BMP-2 upon HAP and Zn-HAPs during the debinding and desorption processes. (0 to 0.1 ns: debinding process; 0.1 to ~10.1 ns: desorption process. A, HAP; B, Zn1-HAP; C, Zn2.5-HAP; D, Zn5-HAP; E, Zn10-HAP.).

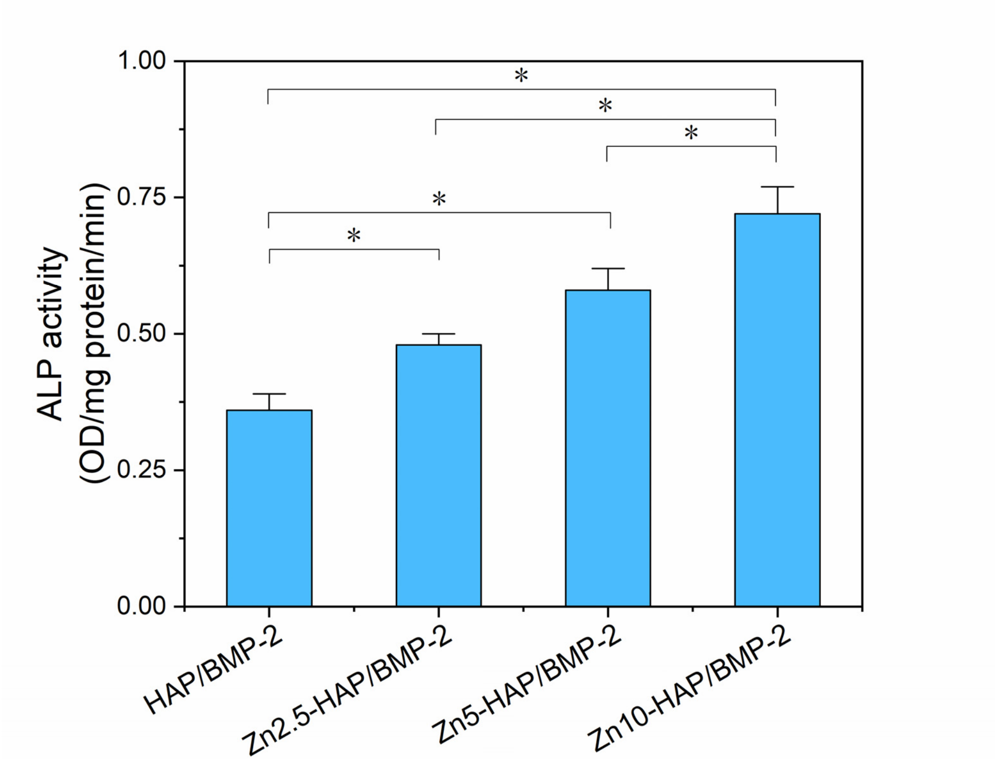

Figure S5. ALP activity of C2C12 cells cultured with HAP/BMP-2 and Zn-HAPs/BMP-2 for 3 days.

*, $p < 0.05$, compared with corresponding samples.
